# Assessing the spatial distribution of cervical spinal cord activity during tactile stimulation of the upper extremity in humans with functional magnetic resonance imaging

**DOI:** 10.1016/j.neuroimage.2020.116905

**Published:** 2020-05-06

**Authors:** Kenneth A. Weber, Yufen Chen, Monica Paliwal, Christine S. Law, Benjamin S. Hopkins, Sean Mackey, Yasin Dhaher, Todd B. Parrish, Zachary A. Smith

**Affiliations:** a Systems Neuroscience and Pain Lab, Department of Anesthesiology, Perioperative and Pain Medicine, Stanford University, Palo Alto, CA, USA; b Department of Radiology, Northwestern University Feinberg School of Medicine, Chicago, IL, USA; c Department of Neurological Surgery, University of Oklahoma Health Sciences Center, Oklahoma City, OK, USA; d Department of Neurological Surgery, Northwestern University Feinberg School of Medicine, Chicago, IL, USA; e Department of Physical Medicine and Rehabilitation, University of Texas Southwestern Medical Center, Dallas, TX, USA

**Keywords:** Functional MRI, Humans, Touch, Sensory function, Spinal cord, Upper extremity

## Abstract

Dermatomal maps are a mainstay of clinical practice and provide information on the spatial distribution of the cutaneous innervation of spinal nerves. Dermatomal deficits can help isolate the level of spinal nerve root involvement in spinal conditions and guide clinicians in diagnosis and treatment. Dermatomal maps, however, have limitations, and the spatial distribution of spinal cord sensory activity in humans remains to be quantitatively assessed. Here we used spinal cord functional MRI to map and quantitatively compare the spatial distribution of sensory spinal cord activity during tactile stimulation of the left and right lateral shoulders (i.e. C5 dermatome) and dorsal third digits of the hands (i.e., C7 dermatome) in healthy humans (n = 24, age = 36.8 ± 11.8 years). Based on the central sites for processing of innocuous tactile sensory information, we hypothesized that the activity would be localized more to the ipsilateral dorsal spinal cord with the lateral shoulder stimulation activity being localized more superiorly than the dorsal third digit. The findings demonstrate lateralization of the activity with the left- and right-sided stimuli having more activation in the ipsilateral hemicord. Contradictory to our hypotheses, the activity for both stimulation sites was spread across the dorsal and ventral hemicords and did not demonstrate a clear superior-inferior localization. Instead, the activity for both stimuli had a broader than expected distribution, extending across the C5, C6, and C7 spinal cord segments. We highlight the complexity of the human spinal cord neuroanatomy and several sources of variability that may explain the observed patterns of activity. While the findings were not completely consistent with our *a priori* hypotheses, this study provides a foundation for continued work and is an important step towards developing normative quantitative spinal cord measures of sensory function, which may become useful objective MRI-based biomarkers of neurological injury and improve the management of spinal disorders.

## 1. Introduction

Spinal conditions affect nearly one billion individuals worldwide and are a leading cause of pain and physical disability globally (Global, 2015; Hoy et al., 2010; Hoy et al., 2014a; Hoy et al., 2014b). Radiculopathy is a common spinal condition resulting from compression and irritation of the spinal nerve roots leading to pain, sensory deficits, muscle weakness, and decreased function (Iyer and Kim, 2016). Sensory testing is commonly used clinically to detect areas of altered sensation and localize neurological injury in spinal conditions. Dermatomal maps provide information on the spatial distribution of the cutaneous innervation of spinal nerves, and dermatomal deficits together with confirmatory examination findings (i.e., segmental motor and reflex deficits), medical imaging, and electro-diagnostic testing can help isolate the level of spinal nerve root involvement and guide clinicians in diagnosis and treatment (Greenberg, 2003).

In practice, however, the clinical presentation of radiculopathy and related spinal conditions is often more complicated due to the frequent presence of multi-level degenerative changes involving multiple spinal nerve root levels and even compression of the spinal cord, itself. While conventional medical imaging (radiographs, computed tomography, and magnetic resonance imaging (MRI)) can provide excellent visualization of the spatial relationship between the neural structures and the spinal anatomy, the predictive value of conventional imaging for spinal disease is limited. Incidental findings of degeneration, disc pathology, and spinal nerve root compromise, as well as spinal cord compression, are present with high prevalence in asymptomatic individuals (Brinjikji et al., 2015; Romeo et al., 2019), and the increased use of medical imaging has not necessarily led to meaningful improvements in clinical outcomes (Deyo et al., 2009; Maus, 2010). More sensitive measures of nervous system pathology are needed to better identify and localize the clinically significant spinal pathology to improve the clinical management of the spine.

New advancements in MRI acquisition and analysis techniques have recently expanded our ability to non-invasively investigate the spinal cord with high-spatial resolution (Alley et al., 2018; Stroman et al., 2014; Wheeler-Kingshott et al., 2014). Advanced structural spinal cord imaging techniques, including diffusion-weighted and magnetization transfer MRI, are showing promise at linking spinal cord pathology to a patient’s clinical state as recently demonstrated in cervical spondylotic myelopathy, multiple sclerosis, amyotrophic lateral sclerosis, and incomplete spinal cord injury (Cloney et al., 2018; Eden et al., 2019; Hopkins et al., 2018; Maki et al., 2018; Martin et al., 2017; Paquin et al., 2018; Smith et al., 2018). Functional MRI provides a means to measure neural activity using the blood oxygenation level dependent (BOLD) contrast and is routinely used clinically for pre-surgical planning to localize brain function in relation to the brain pathology (Nadkarni et al., 2015; Ogawa et al., 1990). Spinal cord fMRI, however, is particularly challenging due to magnetic field inhomogeneities at bone-tissue interfaces causing imaging artifacts with conventional BOLD imaging and the high levels of physiological noise from the cardiac and respiratory cycles, which confound signal detection (Summers et al., 2010). The spinal cord imaging field is gradually overcoming these challenges (Barry et al., 2018), and spinal cord fMRI is now showing potential to localize areas of normal and abnormal sensorimotor function with applications in fibromyalgia, multiple sclerosis, cervical spondylotic myelopathy, and incomplete spinal cord injury (Cadotte et al., 2012; Conrad et al., 2018; Liu et al., 2016; Martucci et al., 2019). Spinal cord fMRI combined with sensory testing may improve our ability to localize the level of spinal nerve root compromise while also providing stronger prognostic and predictive information on the capacity for functional recovery in spinal conditions.

Several studies have used spinal cord fMRI in humans to map and compare the spatial distribution of sensory spinal cord activity at varying stimulation sites (i.e., left versus right and across different dermatomes) using tactile (Lawrence et al., 2008; Stracke et al., 2005; Stroman and Ryner, 2001) and thermal (Cadotte et al., 2012; Nash et al., 2013; Stroman et al., 2012; Stroman et al., 2004; Stroman et al., 2001, Stroman et al., 2002a; Stroman et al., 2002b) stimuli. While demonstrating the feasibility of fMRI for mapping spinal cord sensory activity, only three of these studies quantitively assessed the spatial distribution of the spinal cord activity at either the group or subject level, and of these three studies, none demonstrated that the activity was significantly localized to any specific area of the spinal cord (Lawrence et al., 2008; Stracke et al., 2005; Stroman et al., 2002a). The lack of quantitative spatial analyses limits our ability to interpret the localization of the reported activity, and the spatial distribution of the spinal cord sensory activity captured with fMRI in humans remains largely unknown.

The purpose of this study is to quantitatively assess the spatial distribution of spinal cord activity from left- and right-sided tactile stimulation of the lateral shoulders and dorsal third digits of the hands in healthy human participants at the group and subject level. To accomplish this, we leverage recently available advanced fMRI acquisition (i.e., T_2_*-weighted, reduced field-of-view gradient-echo echo-planar-imaging) and analysis (i.e., the open-source Spinal Cord Toolbox) techniques (De Leener et al., 2017; Rieseberg et al., 2002), which have allowed us to previously demonstrate highly localized spinal cord activity during an upper extremity motor task (Weber et al., 2016b). Based on the laterality of the projections of the primary sensory afferents to the dorsal horn of the spinal cord, we hypothesize that tactile stimulation will result in activity localized primarily to the ipsilateral dorsal spinal cord (Abraira and Ginty, 2013), and considering the somatotopic arrangement of the dermatomal maps, we expect the stimulation of the lateral shoulders to result in activity localized more superiorly in the cervical spinal cord than stimulation of the dorsal third digits (Greenberg, 2003). This work represents an important step towards developing normative quantitative spinal cord measures of sensory function, which may become useful objective MRI-based biomarkers of neurological injury, providing important diagnostic, prognostic, and predictive information for the management of spinal disorders.

## 2. Methods

### 2.1. Participants

Twenty-nine healthy volunteers (7 male and 22 female; average age ± standard deviation (SD) = 36.0 ± 11.8 years) participated in the study. Subjects reported no neuromusculoskeletal diseases or contraindications to MRI. The subjects provided written informed consent, and Northwestern University’s Institutional Review Board (Chicago, IL, USA) approved the study.

### 2.2. Imaging protocol

Imaging was performed using a 3 T Siemens Prisma magnetic resonance scanner with the participants placed supine on the scanner bed. The scanner was equipped with a 64-channel head/neck coil and the head coil elements 5–7 (inferior portion of the head coil) and the anterior and posterior neck coil elements (24 channels) were used to receive the signal. A SatPad™ cervical collar was used to increase the magnetic field homogeneity across the cervical spine and reduce bulk motion during scanning (Maehara et al., 2014). For functional imaging, twenty-five transverse slices of the cervical spinal cord were acquired with a T_2_*-weighted gradient-echo echo-planar-imaging sequence using ZOOMit selective field-of-view imaging (TR_3D_ = 2000 ms, 450 vol, TE = 30 ms, flip angle = 80° slice order = interleaved, field-of-view = 128 × 44 mm^2^, acquisition matrix = 128 × 44, in-plane resolution = 1 × 1 mm^2^, slice thickness = 3 mm, discarded two dummy volumes) (Pfeuffer et al., 2002; Rieseberg et al., 2002). The imaged volume was centered on the spinal cord at the C5 vertebral level. The field-of-view was rotated in the sagittal and coronal planes so the slice plane was orthogonal to the superior-inferior axis of the spinal cord. The choice of imaging sequence and sequence parameters were similar to those used by Weber et al. (2016a, b) and Kinany et al. (2019) (Kinany et al., 2019; Weber et al., 2016a, b). For registration of the functional images to template space, a high-resolution T_2_-weighted structural image of the cervical spine and upper thoracic spine was acquired using a single slab three-dimensional turbo spin echo sequence with a slab selective, variable excitation pulse (SPACE, TR = 1500 ms, TE_EFF_ = 135 ms, echo train length = 74, flip angle = 90°/140°, 64 sagittal slices, 0.8 mm thickness, iPAT acceleration factor = 3, effective in-plane resolution 0.8 × 0.8 mm^2^, interpolated in-plane resolution 0.4 × 0.4 mm^2^) (Lichy et al., 2005; Mugler et al., 2000).

### 2.3. Tactile stimulation protocol

Functional imaging was performed in 15 min runs (450 volumes). During each run, alternating left- and right-sided tactile stimuli were applied manually to the skin over the lateral shoulders (i.e., C5 dermatome) or dorsal third digits of the hands (i.e., C7 dermatome) using Wilbarger therapy brushes attached to wooden dowels. The stimuli were applied at approximately 2 Hz by two examiners in the scanner room. The stimulation runs consisted of 20 trials of 15 s of rest followed by 15 s of left-sided stimulation and 15 s of right-sided stimulation in an pseudorandomized alternating order ((rLR or rRL) × 20, where r = 15 s resting block with no stimulation, L = 15 s left-sided stimulation block, and R = 15 s right-sided stimulation block). One run was performed for each stimulation site (lateral shoulder or dorsal third digit). Instructions for the onset, offset, and side of stimulation were provided to the examiners during scanning by projecting visual cues onto a screen placed inside the scanner room. The participants were unable to see the visual cues to limit their ability to anticipate the onset and offset of the stimulation. For each run, one examiner applied the left-sided stimuli, while the other examiner applied the right-sided stimuli. The lateral shoulder stimuli and the dorsal third digit stimuli were applied at opposite ends of the magnetic bore, near the head and feet, respectively. The order of the stimulation sites (lateral shoulder or dorsal third digit) and examiner assignment (left- or right-sided stimuli) was pseudorandomized across the participants to reduce order effects. Throughout imaging, the participants were instructed to remain still and try not to produce any movements.

### 2.4. Image processing

#### 2.4.1. Motion correction

The Oxford Center for fMRI of the Brain’s (FMRIB) Software Library (FSL) was used for image preprocessing and statistical analyses (Jenkinson et al., 2012; Smith et al., 2004). Motion correction was performed using FMRIB’s Linear Image Registration Tool (FLIRT) with spline interpolation and a normalized correlation cost function in two phases (Jenkinson et al., 2002). First, a manually drawn binary mask limited to the region around the spinal canal was generated (Supplementary Fig. 1). This mask was used to weight the reference image to exclude areas of non-rigid motion outside the spinal column from the respiratory cycle and swallowing. For the first phase of motion correction, the volumes were initially aligned to the middle volume of each run using a three-dimensional rigid body realignment. The mean across the timeseries was then calculated, and motion correction was repeated using the mean image as the reference volume. To correct for slice independent motion due to the non-rigidity of the cervical spine and physiological motion from swallowing and the respiratory cycle, a second phase of motion correction was conducted in which a two-dimensional rigid realignment was performed independently for each axial slice using the mean image from the first phase of motion correction as the reference image (Cohen-Adad et al., 2009; Weber II et al., 2014). As motion correction does not correct for all motion-related noise, motion outlier volumes were identified with FSL’s motion outlier detection tool using the intensity-based DVARS (root mean square variance of the temporal derivative of the time courses) metric and the default threshold (box-plot cutoff = 75th percentile + 1.5 × interquartile range) (Power et al., 2012).

#### 2.4.2. Spatial normalization

The Spinal Cord Toolbox (Version 4.0.1) and PAM50 spinal cord template (resolution = 0.5 × 0.5 × 0.5 mm^3^) were used for spatial normalization of the functional images from native space to standard space (De Leener et al., 2017, 2018). First, the T_2_-weighted structural image was cropped to include the C2 to T1 vertebrae. A spinal cord mask was automatically generated using a convolutional neural network segmentation model (Gros et al., 2019). The C2 and T1 vertebrae were manually identified, and a vertebral landmarks mask was generated. The structural image was straightened along the spinal cord using the binary mask of the spinal cord, and the image was then non-linearly registered to the PAM50 T_2_-weighted spinal cord template (Fonov et al., 2014). The spinal cord was manually segmented from the mean motion corrected functional image. The PAM50 T_2_*-weighted template was then registered to the mean functional image using the template to structural warping field to initialize the registration and the spinal cord segmentation masks. The warping fields from each step of the normalization process were then concatenated allowing for the transformation of the functional images to standard space (Supplementary Fig. 2). The transformed images at each step were visually inspected for quality control. All reported coordinates are in the PAM50 spinal cord template space using the voxel coordinates.

#### 2.4.3. Image denoising

The cardiac and respiratory cycles are sources of noise and can confound signal detection. Therefore, the respiratory and cardiac signals were collected during scanning, and slice specific voxelwise noise regressors were generated using FSL’s physiological noise modeling (PNM) tool, which uses a model-based approach similar to retrospective correction of physiological motion effects (RETROICOR) (Brooks et al., 2008; Glover et al., 2000). In brief, a cardiac phase and respiratory phase were assigned to each slice, and the cardiac and respiratory signals were then modeled using sine and cosine terms with the principal frequency and the next three harmonics (16 regressors). Multiplicative terms were included to account for the interaction of the cardiac and respiratory cycles (16 additional regressors). Slice specific CSF regressors were also generated by extracting the first five principal components of the CSF signal using a manually drawn mask of the spinal canal and AFNI (3dpc, Supplementary Fig. 1) (Cox, 2012). The time series was high pass filtered (cutoff = 100 s), and then the high pass filtered noise regressors (i.e., cardiac, respiratory, and CSF) were regressed from the motion corrected functional time series using FSL’s Improved Linear Model (FILM) (Worsley et al., 2001). The denoised time series was then slice-timing corrected and warped to PAM50 template space. The spinal cord was then extracted using the PAM50 spinal cord mask, and the images were spatially smoothed using FSL’s SUSAN with a 2 mm^3^ full width half maximum (FWHM) Gaussian smoothing kernel (Smith and Brady, 1997).

#### 2.4.4. Subject and group level analyses

Statistical maps of the preprocessed times series were generated for each run using FILM with prewhitening (Woolrich et al., 2001). The stimuli were modeled using trialwise hemodynamic response function (gamma, phase 0 s, standard deviation 3 s, average lag 6 s) convolved vectors for the left-sided (20 trials) and right-sided (20 trials) tactile stimuli as explanatory variables. The temporal derivatives of the tactile stimulation vectors, the six motion parameters from the first phase of motion correction, and the motion outlier volume regressors were included as covariates of no interest. Subject level average activation maps for the left- and right-sided stimuli and associated contrasts (left > right and right > left) were then generated in a second-level fixed effects analysis. *A priori* FMRIB’s Local Analysis of Mixed Effects (FLAME) Stage 1 was planned to be used to generate group level activation maps (Beckmann et al., 2003; Woolrich, 2008; Woolrich et al., 2004). However, significant group level activation was not consistently present with a mixed effects analysis, and instead, the reported group level activation was generated from a fixed effects analysis (See Results and Discussion). Voxels with a Z-score > 2.3 (p < 0.01, uncorrected) were considered active at the subject level. No correction for multiple comparisons was performed at the subject level due to the smaller volume of interrogation. Group level activation was defined using a voxelwise threshold of Z-score > 2.3 with a cluster significance threshold of p < 0.05 to correct for multiple comparisons. The number of active voxels and the average Z-score of the active voxels at the group and subject level were compared across the stimulation sites and contrasts. Group level analyses were confined to the region of intersection of the subject level functional images.

### 2.5. Spatial analysis

The spatial localization of the activity was quantified by counting the number of active voxels in the left, right, dorsal, and ventral hemicords and the C5, C6, and C7 spinal cord segment levels at the group and subject level for each stimulation site and side of stimulation. To summarize the localization of the activity at the subject level, left-right (LR) and dorsal-ventral (DV) indices were calculated by dividing the difference in the number of active voxels between the respective hemicords by the sum (number of active voxels in the entire spinal cord) for each stimulation site and side of stimulation (Seghier, 2008). For the LR index, a value of +1.0 indicates that all active voxels were in the left hemicord while a value of −1.0 indicates that all active voxels were in the right hemicord. For the DV index, a value of +1.0 indicates that all active voxels were in the dorsal hemicord while a value of −1.0 indicates that all active voxels were in the ventral hemicord. To assess the localization of the activity along the superior-inferior axis of the spinal cord, the center-of-gravity (COG) for the left- and right-sided activity was calculated along the superior-inferior axis (z-axis) at the group and subject level and then compared between the lateral shoulder and dorsal third digit stimulation. The localization of the activity to the gray matter and white matter was also assessed. As the volume of the white matter is more than three times the volume of the gray matter, the ratio of the percentage of gray matter activation to the percentage of white matter activation was calculated to account for the differences in volume. The localization of the gray matter and white matter was assessed at the group and subject level. To assess the subject level variability in the location of activity, the subject level activation maps for each stimulation site and contrast were binarized (Z-score > 2.3, uncorrected), and consistency maps showing the spatial overlap in activity across the participants were generated.

### 2.6. Time-dependent effects

Spinal cord responses to repeated stimuli may habituate (i.e., decrease) or sensitize (i.e., increase) over time, and time-dependent changes in the response to the stimuli may be an additional source of variability (Bensmaia et al., 2005; Cevik, 2014). To investigate time-dependent changes in spinal cord activity, the stimulation run was divided into five sets of four consecutive trials. The average number of active voxels and the average Z-score of the active voxels for each set were averaged for each stimulation site and contrast, and the presence of a linear change in the response to the repeated stimuli was assessed.

### 2.7. Statistical testing

For all non-imaging statistical tests, IBM SPSS Statistics for Windows Version 25 (IBM Corp., Armonk, NY, USA) was used, and an α < 0.05 was used as the threshold for statistical significance.

## 3. Results

**Fig. 1.**
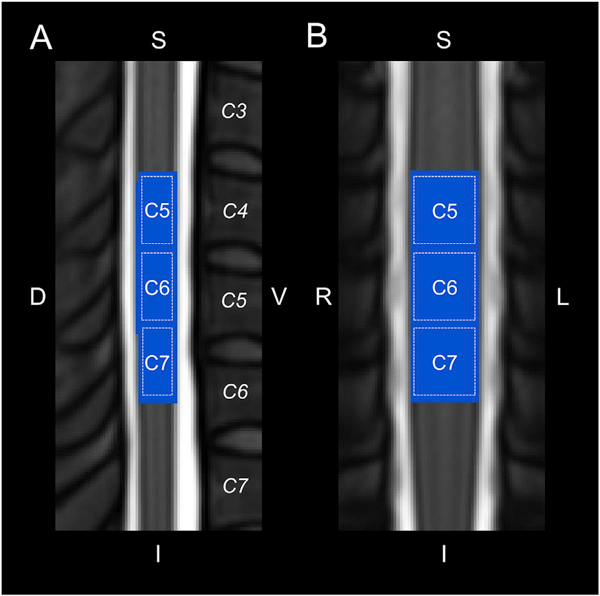
Legend showing the approximate location of the C5, C6, and C7 spinal cord segments in relation to the spinal cord vertebrae (italics) on the PAM50 T_2_-weighted spinal cord template. Midsagittal (A) and midcoronal (B) slices are shown. The spinal cord segments were identified using the spinal cord segment probability maps included in the Spinal Cord Toolbox. The functional imaging spanned 25 3 mm thick axial slices centered at the C5 vertebra. The blue region corresponds to the group mask generated from the intersection of the functional images across the participants. Group level analyses were restricted to the blue region. S = superior, I = inferior, D = dorsal, V = ventral, L = left, R = right.

Of the twenty-nine participants, four participants were excluded for motion artifacts, and one participant was excluded due to a misprescribed field-of-view (centered at C7 vertebra instead of C5 vertebra). Results are reported on the remaining twenty-four participants (5 male and 19 female; average age = 36.8 ± 11.8 years). The intersection of the subject level functional images spanned the C5, C6, and C7 spinal cord segments (Fig. 1), covering the hypothesized distribution of activity for the lateral shoulder (i.e., C5 dermatome) and dorsal third digit (i.e., C7 dermatome) stimulation. Following motion correction, slice-timing correction, and temporal filtering, the average temporal signal-to-noise ratio (TSNR) standard error (SE) over the spinal cord was 24.3 ± 0.4 au and 24.4 ± 0.3 au for the lateral shoulder and dorsal third digit stimulation runs, respectively (Supplementary Fig. 3). After spatial smoothing, the average TSNR was 47.1 ± 1.0 au and 47.2 ± 0.9 au for the lateral shoulder and dorsal third digits stimulation runs, respectively. The average TSNR did not significantly differ between the lateral shoulder and dorsal third digit stimulation runs before (two-tailed paired *t*-test, t = −0.640, p = 0.528) or after spatial smoothing (t = −0.324, p = 0.749), and the average TSNR did not significantly differ across the C5, C6, and C7 spinal cord segments for either the lateral shoulder (repeated measures ANOVA, F = 1.254, p = 0.295) or the dorsal third digit (F = 1.724, p = 0.190) stimulation runs. The mean absolute motion was 0.72 ± 0.07 mm and 0.69 ± 0.07 mm for the lateral shoulder and dorsal third digit stimulation runs, respectively, and the mean relative motion was 0.30 ± 0.02 mm and 0.31 ± 0.01 mm for the lateral shoulder and dorsal third digit stimulation runs, respectively. Neither the mean absolute motion (two-tailed paired *t*-test, t = 0.446, p = 0.660) nor the mean relative motion (t = −0.867, p = 0.395) significantly differed between the runs.

### 3.1. Group level activity

**Fig. 2.**
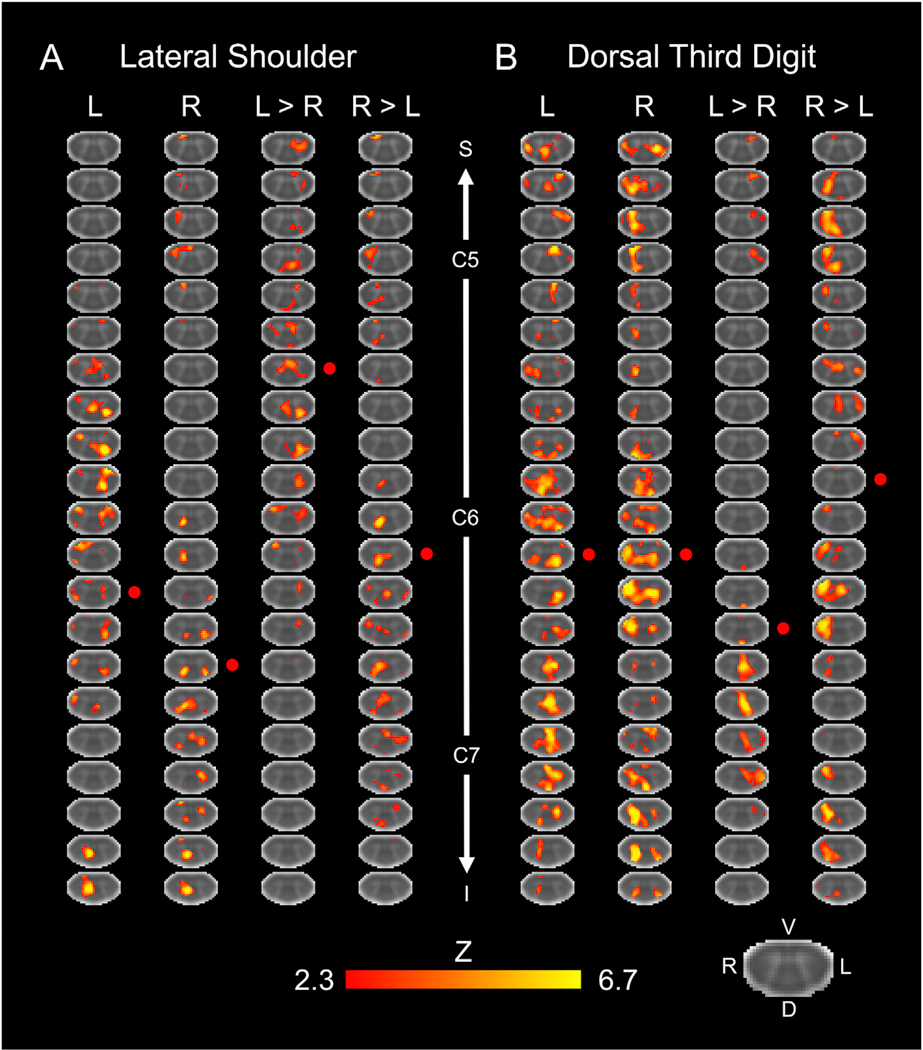
Group level activity for the lateral shoulder (A) and dorsal third digit stimulation (B). Activity is shown for the left (L), right (R), and L > R, and R > L stimulation contrasts. The approximate locations of the centers of the C5, C6, and C7 spinal cord segments are shown. The red circles indicate the approximate center-of-gravity of the activity along the superior-inferior axis. Every 4th axial slice from the intersection of the subject level functional images is shown. The activation maps were generated from a fixed effects analysis at the group level and were voxel-wise thresholded at a Z-score > 2.3 with a cluster-level corrected threshold of p < 0.05. The background image is the PAM50 T_2_*-weighted spinal cord template. S = superior, I = inferior, D = dorsal, V = ventral, L = left, R = right.

**Fig. 3.**
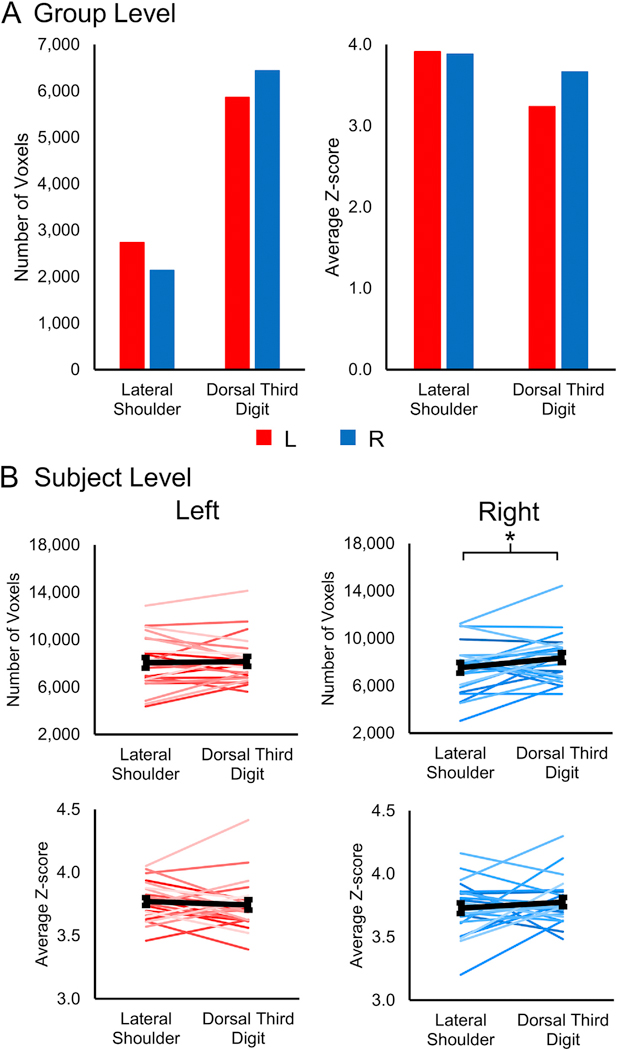
The number of active voxels and average Z-score of the active voxels between the lateral shoulder and dorsal third digit stimulation at the group level (A) and subject level (B) for the left- and right-sided stimuli. Group level activity was from a fixed effects analysis and defined using a voxel-wise threshold of Z-score > 2.3 with a cluster correction for multiple comparisons of p < 0.05. Subject level activity was defined using a voxel-wise threshold of Z-score > 2.3 with no correction for multiple comparisons. The average number of active voxels and the average Z-score of the active voxels across the participants are shown in black. Error bars = standard error. *p < 0.05.

**Fig. 4.**
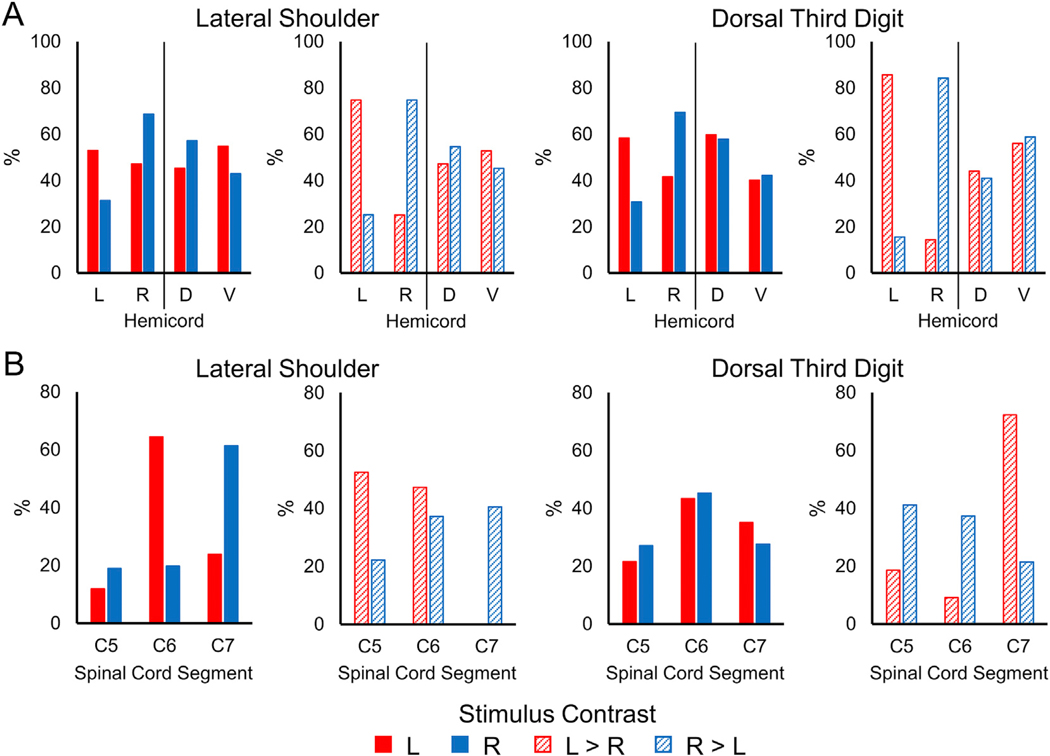
Location of the group level activity for the lateral shoulder and dorsal third digit stimulation. A) The percentage of the group activity localized to the left (L) and right (R) hemicords or the dorsal (D) and ventral (V) hemicords for each of the stimulation contrasts. B) The percentage of the group activity localized to the C5, C6, and C7 spinal cord segments for each of the stimulation contrasts. Group level activity was from a fixed effects analysis using a voxel-wise threshold of Z-score > 2.3 and a cluster-level corrected threshold of p < 0.05.

Using the *a priori* mixed effects analysis, group level spinal cord activity was not consistently present. Only the dorsal third digit stimulation for the right-sided stimuli and the right > left contrast demonstrated significant activation in the mixed effects analysis (Supplementary Fig. 4). The following group level activity results were generated from a fixed effects analysis, which limits the generalizability of the group level findings (Fig. 2). Within the study sample, the spatial extent of the activation at the group level was greater for the dorsal third digit stimulation (5860 and 6434 active voxels for the left- and right-sided stimuli, respectively) compared to the lateral shoulder (2735 and 2137 active voxels for the left- and right-sided stimuli, respectively). However, the average Z-score of the active voxels did not vary considerably between the lateral shoulder (3.91 and 3.88 average Z-score for the left- and right-sided stimuli, respectively) and dorsal third digit (3.24 and 3.66 average Z-score for the left- and right-sided stimuli, respectively) stimulation. Within each stimulation site, the number of active voxels and the average Z-score of the active voxels did not vary considerably between the left-and right-sided stimuli (Fig. 3A). As hypothesized, the group level activity was consistently more localized to the ipsilateral hemicord. The left-sided stimuli and the left > right contrast had more active voxels in the left hemicord for both the lateral shoulder and dorsal third digit stimulation. Likewise, the right-sided stimuli and the right > left contrast had more active voxels in the right hemicord for both stimulation sites. However, the group level activity was not consistently localized to the dorsal hemicord across the stimulation sites and contrasts (Fig. 4A). Additionally, the spatial distribution of the group level activity along the superior-inferior axis was not localized to the C5 or C7 spinal cord segments for either the lateral shoulder or dorsal third digit stimulation, respectively. Instead, the group level activation maps demonstrated a broader distribution of activity along the superior-inferior axis (Fig. 4B). At the group level, across all stimulation sites and contrasts, the ratio of the percentage of gray matter activation to the percentage of white matter activation was greater than one indicating some specificity of the activity to the gray matter (Table 1A).

### 3.2. Subject level activity

At the subject level, spinal cord activity was present for all participants and all stimulation sites and contrasts. The average percent signal change ± SE for the lateral shoulder stimulation was 0.71 ± 0.02% and 0.69 ± 0.03% for the left- and right-sided stimuli respectively. For the dorsal third digit stimulation, the average percent signal change was 0.71 ± 0.03% and 0.68 ± 0.02% for the left- and right-sided stimuli, respectively.

For the lateral shoulder stimulation, the average number of active voxels SE was 8053.3 ± 449.1 and 7516.3 ± 427.0 for the left- and right-sided stimuli, respectively, and the average Z-score of the active voxels SE for the left- and right-sided stimuli was 3.77 ± 0.03 and 3.73 ± 0.04, respectively. For the dorsal third digit stimulation, the average number of active voxels for the left and right-sided stimuli was 8147.7 ± 395.3 and 8345.2 ± 404.2 for the left- and right-sided stimuli, respectively, and the average Z-score of the active voxels for the left- and right-sided stimuli was 3.74 ± 0.04 and 3.78 ± 0.04, respectively. The number of active voxels was signi_fi_cantly greater for the right-sided dorsal third digit stimulation compared to the right-sided lateral shoulder stimulation (two-tailed paired *t*-test, t = −2.236, p = 0.035) but not for the left-sided stimuli (t = −0.324, p = 0.749). The average Z-score of the active voxels did not significantly differ between the lateral shoulder and dorsal third digit stimulation for either the left- (two-tailed paired *t*-test, t = 0.767, p = 0.451) or right-sided (t = 0.967, p = 0.344) stimuli (Fig. 3B). Within each stimulation site, no significant differences in the number of active voxels or the average Z-score of the active voxels were present between the left- and right-sided stimuli (Supplementary Fig. 5). The number of active voxels and average Z-score of the active voxels for the left- and right-sided stimuli were not significantly correlated with the mean absolute motion or mean relative motion for either the lateral shoulder or dorsal third digit stimulation (two-tailed Pearson correlations, p > 0.05).

**Fig. 5.**
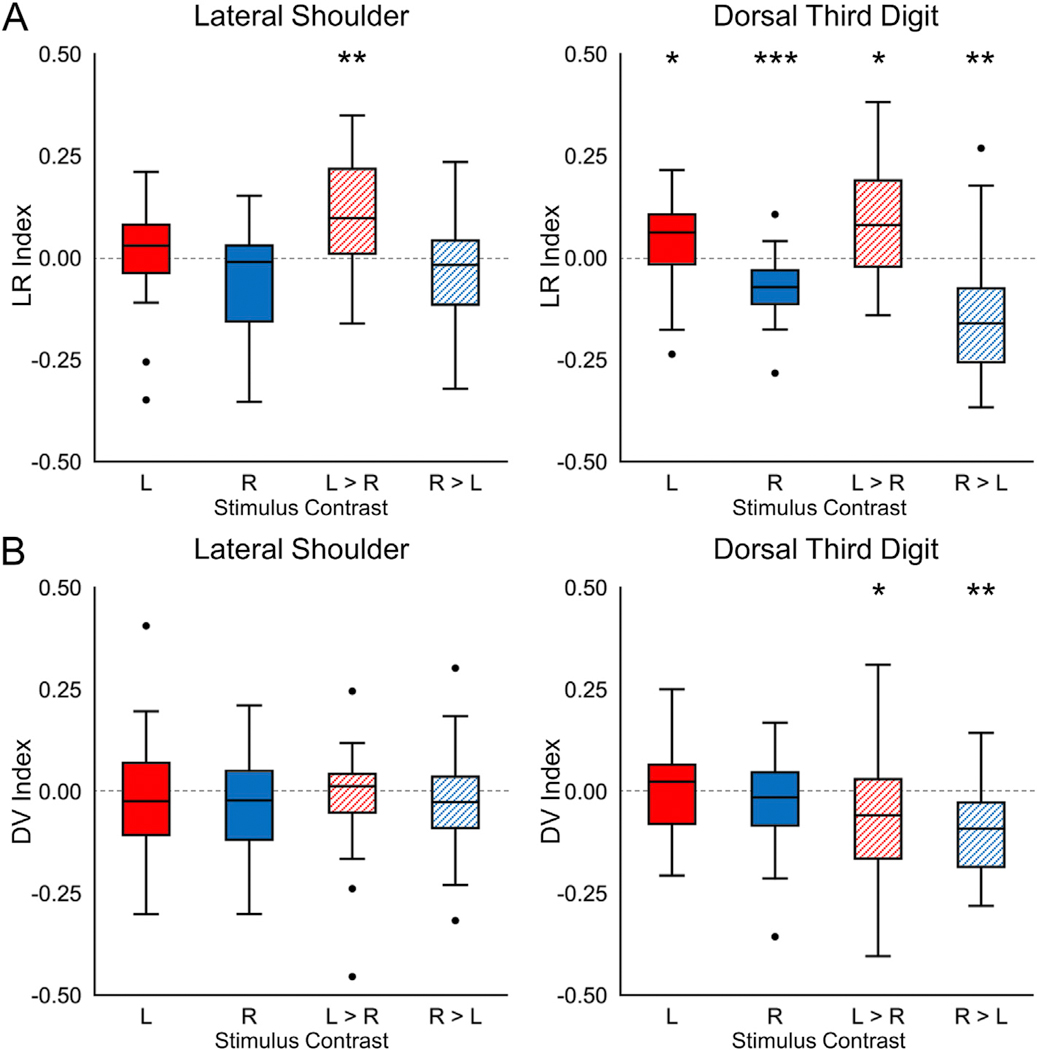
Box plots showing the location of the subject level activity for the lateral shoulder and dorsal third digit stimulation. A) The localization of the subject level activity to the left (L) and right (R) hemicords was summarized using the left-right (LR) index. For the LR index, a value of +1.0 indicates that all active voxels were in the left hemicord while a value of −1.0 indicates that all active voxels were in the right hemicord. B) Similarly, the localization of the subject level activity to the dorsal and ventral hemicords was summarized using the dorsal-ventral (DV) index. For the DV index, a value of +1.0 indicates that all active voxels were in the dorsal hemicord while a value of −1.0 indicates that all active voxels were in the ventral hemicord. The LR and DV indices are shown for each stimulation contrast. Subject level activity was defined using a voxel-wise threshold of Z-score > 2.3 with no correction for multiple comparisons. *p < 0.05, **p < 0.01, ***p < 0.001.

**Fig. 6.**
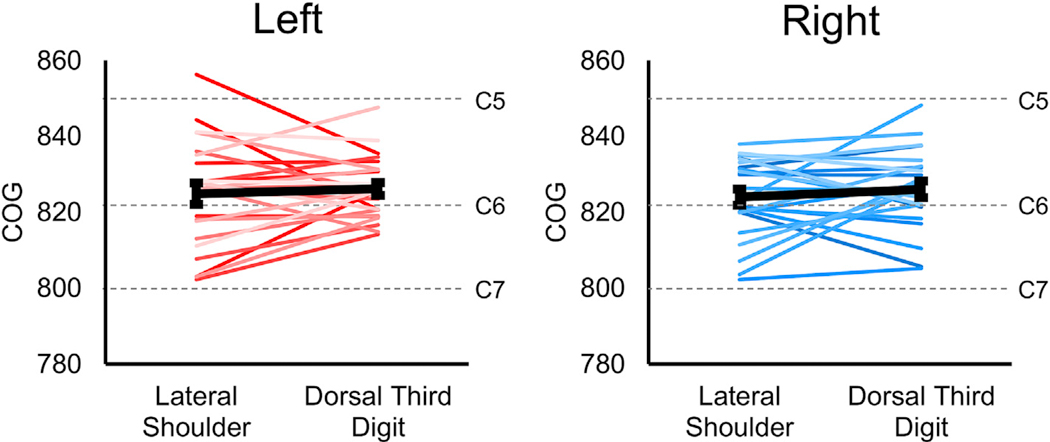
Localization of the subject level activity along the superior-inferior axis. The weighted superior-inferior center-of-gravity (COG) was calculated from the subject level activation maps and compared to the lateral shoulder and dorsal third digit stimulation for the left- and right-sided stimuli. No significant difference in the location of the activity along the superior-inferior axis was demonstrated. The dashed gray lines show the approximate locations of the centers of the C5, C6, and C7 spinal cord segments. The average COG across the participants are shown in black. The COG units are in the voxel coordinates of the PAM50 template. Subject level activity was defined using a voxel-wise threshold of Z-score > 2.3 with no correction for multiple comparisons. Error bars = ± standard error.

For the lateral shoulder stimulation, significant localization of the activity to the ipsilateral hemicord was only present for the left > right contrast (Wilcoxon signed-rank test, hypothesized median = 0). In contrast, significant localization of the activity to the ipsilateral hemicord for the dorsal third digit stimulation was present for both the left- and right-sided stimuli as well as the left > right and right > left contrasts (Fig. 5A and Table 2A). No significant localization of the activity to the dorsal or ventral hemicords was present for the lateral shoulder stimulation. For the third digit stimulation, the activity was only significantly localized for the left > right and right > left contrasts, which demonstrated more ventral hemicord activity (Fig. 5B and Table 2A). Across the participants, the average COG ± SE along the superior-inferior axis for the left-sided stimuli was 824.9 ± 2.8 and 826.2 ± 1.7 for the lateral shoulder and dorsal third digit stimulation, respectively. For the right-sided stimuli, the average COG was 824.0 ± 2.8 and 826.1 ± 2.1 for the lateral shoulder and dorsal third digit stimulation, respectively. The COG for lateral shoulder and dorsal third digit stimulation did not differ across the participants for either the left- (t = −0.558, p = 0.582) or right-sided (t = −0.905, p = 0.375) stimuli (Fig. 6). The COG units are in PAM50 voxel coordinates, and a greater value indicates more superior in the spinal cord. Across the participants, the activity was not significantly localized more to the gray matter or white matter for any of the stimulation sites or contrasts (Wilcoxon signed-rank test, hypothesized median = 1, Table 1B). From the consistency maps, the maximum overlap of the subject level activation maps across the stimulation sites and contrasts was only 14 of the 24 participants, indicating considerable intersubject variability in the spatial localization of the activity across the spinal cord (Supplementary Fig. 6).

### 3.3. Time-dependent

No significant increase or decrease in the number of active voxels or the average Z-score of the active voxels was present for the lateral shoulder or the dorsal third digit stimulation for either the left- or right-sided stimuli (repeated measures ANOVA with linear contrast, p > 0.05, Supplementary Fig. 7).

## 4. Discussion

Here we used fMRI to map and quantitatively compare the spatial distribution of sensory spinal cord activity from tactile stimulation of the upper extremity at different stimulation sites in healthy humans. Tactile stimuli were applied in a block design to the non-glabrous (hairy) skin of the left and right lateral shoulders and dorsal third digits of the hands. This experimental design was chosen to assess the spatial distribution of the activity because the central sites for processing of innocuous tactile sensory information are well known in humans allowing for strong a *priori* hypotheses on the location of the activity. We hypothesized that the activity would be localized more to the ipsilateral dorsal spinal cord with the lateral shoulder stimulation activity being localized more superiorly than the dorsal third digit stimulation activity. The findings demonstrate lateralization of the activity at the group and subject level with the left- and right-sided stimuli having more activation in the respective ipsilateral hemicord. The laterality was most striking for the dorsal third digit stimulation, which demonstrated significant localization of the activity to the ipsilateral hemicord across the stimulation contrasts at the subject level. Contradictory to our hypotheses, the activity for both stimulation sites was not localized more dorsally but spread across the dorsal and ventral hemicords at the group and subject level, and the lateral shoulder and dorsal third digit stimulation activity did not demonstrate a clear superior-inferior localization to their respective spinal cord segments. Instead, the activity for both stimuli had a broader than expected distribution that extended across the C5, C6, and C7 spinal cord segments. In the following, we highlight the complexity of the human spinal cord neuroanatomy and several sources of variability that may explain the observed patterns of activity. We then describe steps forward to improve our ability to map sensory activity in the spinal cord with fMRI and develop clinically useful objective MRI-based biomarkers of sensory function.

Innocuous tactile stimuli of the non-glabrous skin are transduced primarily by hair follicles associated with low-threshold mechanoreceptors and their specialized end organs. These signals are then conveyed via Aβ-, Aδ-, and C-fibers to specific central targets mainly in the dorsal column pathway. In humans, two main central projection pathways exist for innocuous tactile sensory information. In the direct pathway, a subset of low-threshold mechanoreceptors after entering the dorsal horn project axonal branches superiorly via the ipsilateral dorsal column fasciculi making monosynaptic connections to neurons in the ipsilateral dorsal column nuclei in the brainstem. In the indirect pathway, low-threshold mechanoreceptors first synapse on interneurons within the ipsilateral dorsal horn, and then the sensory information is carried to the ipsilateral dorsal column nuclei indirectly via postsynaptic dorsal column neurons (Abraira and Ginty, 2013). Within the spinal cord, the primary sensory afferent projections are also somatotopically organized; sensory afferents from superior regions project to more superior spinal cord segments, while sensory afferents from inferior regions project to more inferior spinal cord segments (Brown and Fuchs, 1975; Wilson et al., 1986).

While evidence for lateralization of the activity to the ipsilateral hemicord was present, contralateral activity was always seen at the group and subject level. This finding is congruent with our previous sensory study, using similar imaging and analysis techniques, that identified contralateral activity during right-sided ventral forearm thermal stimulation (Weber et al., 2016a). The interneuronal networks within the spinal cord actively integrate and modulate the neural activity between hemicords. A common example is the crossed extensor reflex, where, albeit, noxious primary afferents activate ipsilateral interneurons that in turn excite extensor and inhibit flexor motoneurons in the contralateral hemicord (Laflamme and Akay, 2018). Additionally, the propriospinal circuitry contains crossed interneurons, which modulate sensorimotor processing between hemicords and have a known role in interlimb coordination (Juvin et al., 2005). Evidence for the coordinated processing of neural information between the hemicords has recently been identified in humans with fMRI. At 7 T, Barry et al. (2014) identified the presence of bilateral sensory (i.e., left and right dorsal horn connectivity) and motor (i.e., left and right ventral horn connectivity) spinal cord networks at rest in humans (Barry et al., 2014). These networks have now been reproduced at 3 T by independent groups (Eippert et al., 2017b; Weber et al., 2018) and may be altered by spinal cord pathology (Conrad et al., 2018). Given the crossed spinal cord circuitry and recent spinal cord fMRI findings, the presence of contralateral activity in this study may represent the contralateral processing of sensory signals, perhaps for coordination of bilateral sensorimotor function, and may be expected under normal conditions.

Ventral activity was also not expected as low-threshold mechanoreceptors project mainly to the dorsal horn and do not make monosynaptic connection to motoneurons. These afferents, however, do synapse on dorsal horn interneurons, which then project to motoneurons in the ventral horn, and interneuronal processing could also explain the ventral activation (Abraira and Ginty, 2013). During the runs, participants were asked to remain still and not move in response to the stimulation. While no obvious movements of the upper extremity were noticeable during the study, the participants may have maintained a low-level reflexive (e.g., tickle) or voluntary (e.g. muscle tensing in response to the stimuli) contraction of the upper extremity during the sensory stimulation, and, if present, this could further explain the ventral activity (Pritchard, 1933). The use of electromyography could have identified the possible muscle activity during the experiment, and the electromyography signal could have been included as covariate in the general linear model to account for this source of noise. Monitoring muscle activity during spinal cord fMRI experiments is recommended in future studies.

The broad and overlapping superior-inferior distribution of activity for both stimulation sites may also reflect the complexity of interneuronal processing and the distributed projections of primary afferents. Upon entering the cord, low-threshold mechanoreceptor axons (primarily Aβ-fibers) may ascend or descend multiple spinal cord segments while sprouting collaterals creating a longitudinal column of terminal arborizations (Brown et al., 1977; Li et al., 2011). In addition, the ascending and descending propriospinal circuits integrate sensorimotor neural activity across multiple spinal cord segments (Frigon, 2017), and intersegmental anastomoses between spinal nerve roots are a common anatomical variant, which would further disrupt the correspondence between the spinal nerve root and spinal cord segment levels (Moriishi et al., 1989). Taken together, stimulation of a single site in this study likely leads to activity across multiple spinal cord levels possibly explaining the lack of localized activity to a single spinal cord segment for either the lateral shoulder (i.e., C5 spinal cord segment) or dorsal third digit (i.e., C7 spinal cord segment) stimulation.

Dermatomal maps are a mainstay of clinical practice providing information on the spatial distribution of the cutaneous innervation of spinal nerves and are used to localize the level of spinal nerve root involvement in neurological injury (Greenberg, 2003). However, the accuracy of the dermatomal maps has been called into question, and the classical dermatomal maps do not show any overlap between adjacent spinal cord segments, assume left-right symmetry, and fail to provide any information on interindividual variability (Lee et al., 2008). Considering this, consistent stimulation of specific dermatomes across the participants is highly unlikely. Additionally, considerable variability exists in the location of the spinal cord segments across individuals (Cadotte et al., 2015), which is not taken into account by the spatial normalization methods employed in this study. Both the interindividual variability in the cutaneous segmental innervation and the location of spinal cord segments likely contributed to the high spatial variability in the activity seen across the participants and the absence of consistent group level activity with a mixed effects analysis. The use of a fixed effects analysis is a limitation and reduces the generalizability of the group level findings. Improved spatial normalization algorithms that account for the variability in the spinal cord segment levels may help improve the agreement of the spinal cord neuroanatomy between participants and the analysis of the spatial localization of activity. Additionally, the generation of normative activation maps in a larger sample (n > 100) would provide a probabilistic distribution of the activity for stimulation sites and measures of interindividual variability. With a larger sample, the probabilistic maps may demonstrate evidence of a superior-inferior distribution of activity that corresponds more closely to the classical dermatomal maps.

Another source of variability, which may have confounded the comparison of the lateral shoulder and dorsal third digit activity, is the differential distribution in the density of mechanoreceptors between the two stimulation sites. Spatial acuity is the ability to distinguish between two stimuli close in space and is a function of the innervation density of the tissue (Mancini et al., 2014). Tactile spatial acuity and the density of mechanoreceptors vary across different body sites, and both tend to increase proximally to distally in the extremities (Cody et al., 2008; Johansson and Vallbo, 1979). The spatial acuity to tactile stimuli is much greater at the dorsum of the hand than at the shoulder (Mancini et al., 2014). Based on the difference in spatial acuity across the stimulation sites, tactile stimulation of the dorsal third digit likely resulted in the activation of a greater number of mechanoreceptors leading to a greater sensory input into the spinal cord than the lateral shoulder stimulation, which may explain the greater spatial extent of the dorsal third digit stimulation activity seen at the group level. In hindsight, stimulation of the lateral (i.e., C6 dermatome) and medial (i.e., C8 dermatome) dorsal hands may have allowed us to still study the superior-inferior distribution of the activity with less discrepancy in mechanoreceptor density between the stimulation sites.

Low-threshold mechanoreceptors are classified based on their conduction velocities, adaptation properties, and associated cutaneous end organs. The combination of these factors results in response properties that are tuned to specific sensory features and the transmission of this information to central sites (Abraira and Ginty, 2013). In this study, tactile stimuli were delivered manually at approximately 2 Hz. We chose a dynamic tactile stimulus versus a static tactile stimulus (e.g., sustained pressure) in order to maintain a constant neural input into the dorsal column pathways over the block of stimulation. The dynamic brush stimuli should have resulted in repeated hair follicle deflection and sustained phasic firing of the rapidly adapting low-threshold mechanoreceptors, while the maintained pressure of the skin should cause sustained firing of the slowly adapting Merkel cells (Abraira and Ginty, 2013). Although, the stimulus likely provided sustained neural responses across the stimulation blocks, we did not control the pressure or frequency of the stimulus, and slight variation in the site of stimulation within a single run was also likely. The potential variability in the delivery of the stimuli possibly resulted in fluctuations in the BOLD signal over the run. The trialwise analysis, in which each stimulus block was modeled separately, was employed to be more flexible and allow for slight changes in the timing and amplitude of the delivery of the stimuli. We are currently developing computerized stimulation devices that can deliver calibrated and controlled tactile stimuli to limit the variability in the mechanoreceptor activation. The computerized stimulation devices will also provide a means to assess multiple stimulation sites within a single run, increasing the efficiency of the experimental design and permitting more direct comparisons between sites. Spinal cord responses to repeated stimuli may also habituate or sensitize over time, which could be another source of variability. However, no significant time-dependent changes in the activity were seen, suggesting that the spinal cord activity at large remained consistent over the course of the experiment.

Compared to brain fMRI, spinal cord fMRI is still in its early stages. For example, tools for registration and spatial normalization to a standard template, such as the Spinal Cord Toolbox and the PAM50 template used in this study, have only recently been available for spinal cord MRI analysis (De Leener et al., 2017, 2018). Spinal cord fMRI has its own unique issues compared to the brain, and most of the preprocessing steps used in this study remain to be thoroughly investigated and optimized. The non-rigidity of the spinal cord, for instance, leads to non-uniform displacements of the spinal cord during the respiratory cycle, which cannot be corrected for with standard rigid-body motion correction used in brain fMRI (Cohen-Adad et al., 2009; Weber II et al., 2014). Physiological noise removal and spatial smoothing are other processing steps that require further attention by the spinal cord fMRI field (Eippert et al., 2017a; Weber II et al., 2017). We plan to use the current dataset to systematically investigate the preprocessing and analysis steps to increase the sensitivity and specificity of spinal cord fMRI to detect sensory activity. Recently, methods for simultaneous functional imaging of the spinal cord and brain in humans have been developed (Finsterbusch et al., 2013; Islam et al., 2019; Vahdat et al., 2015). Considering the somatotopic organization of sensory processing in the brain, simultaneous spinal cord-brain fMRI may further improve our ability to differentiate sensory activity between stimulation sites (Grodd et al., 2001; Penfield and Boldrey, 1937; Qi and Kaas, 2006). Additionally, adaptation aftereffects are a phenomenon where the presentation of one stimulus interferes with the perception of a following stimulus (Calzolari et al., 2017). Although the perception of the stimulation was not assessed in the present study, adaptation aftereffects could have led to interference in the activity between the alternating pairs of left- and right-sided stimulation. As both spinal and supraspinal mechanisms have been presumed to contribute to this phenomenon, simultaneous spinal cord-brain fMRI may also help disentangle the influence of supraspinal processing on spinal tactile activity (i.e., descending modulation) and better shape our understanding of the interaction between spinal and supraspinal processes and sensory perception. Finally, in a larger more generalizable sample, we intend to use these more optimized imaging and analysis methods in combination with multivariate machine-learning methods to develop models of normal sensory function similar to the recent motor study by Kinany et al. (2019) (Kinany et al., 2019). These models could then be applied to patients with spinal conditions and known sensory deficits to see whether the spinal cord activity can provide additional diagnostic, prognostic, and predictive information on the level of spinal nerve root injury.

Here we mapped and quantitatively compared the spatial distribution of sensory spinal cord activity at different upper extremity stimulation sites in healthy humans. While the findings were not completely consistent with our *a priori* hypotheses, this study provides a foundation for continued work. Future work will use better controlled sensory stimuli, optimized imaging and analysis methods, and a larger more generalizable sample to generate probabilistic maps of spinal cord activity at multiple stimulation sites. This an important step towards developing normative quantitative spinal cord measures of sensory function, which may become useful objective MRI-based biomarkers of neurological injury and improve the management of spinal disorders.

## Supplementary Material

1

## Figures and Tables

**Table 1:** 

A. Group level localization of activity to gray matter (GM) and white matter (WM)			

Lateral Shoulder			
Contrast	% GM Activated	% WM Activated	% GM/% WM

L	15.5	10.4	1.50
R	31.6	22.7	1.39
L > R	13.3	7.8	1.71
R > L	34.8	24.9	1.40
Dorsal Third Digit			
Contrast	% GM Activated	% WM Activated	% GM/% WM

L	12.8	8.7	1.46
R	8.4	7.0	1.20
L > R	10.2	8.7	1.17
R > L	20.2	17.1	1.18

B. Subject level localization of activity to GM and WM					

Lateral Shoulder					
Contrast	Number of subjects with % GM/% WM > 1	Median	IQR	Z-score	P-value

L	10	0.973	0.201	−1.000	0.317
R	11	0.970	0.248	−0.229	0.819
L > R	14	1.009	0.312	0.314	0.753
R > L	7	0.859	0.233	−1.829	0.067
Dorsal Third Digit					
Contrast	Number of subjects with % GM/% WM > 1	Median	IQR	Z-score	P-value

L	14	1.041	0.299	0.371	0.710
R	11	0.988	0.263	0.629	0.530
L > R	9	0.929	0.294	−0.286	0.775
R > L	16	1.043	0.278	1.571	0.116

L = left, R = right, D = dorsal, V = ventral, IQR = interquartile range.

**Table 2:** 

A. Localization of activity to the left or right hemicords					

Lateral Shoulder					
Contrast	Number of subjects with LR index > 0	Median LR Index	IQR	Z-score	P-value

L	16	0.030	0.119	1.314	0.189
R	11	−0.010	0.186	−1.514	0.130
L > R	20	0.097	0.208	3.114	**0.002**
R > L	9	−0.017	0.157	−1.000	0.317
Dorsal Third Digit					
Contrast	Number of subjects with LR index > 0	Median LR Index	IQR	Z-score	P-value

L	17	0.062	0.122	2.143	**0.032**
R	3	−0.072	0.083	−3.543	< **0.001**
L > R	16	0.080	0.212	2.514	**0.012**
R > L	5	−0.161	0.181	−3.171	**0.002**

B. Localization of activity to the dorsal and ventral hemicords					

Lateral Shoulder					
Contrast	Number of subjects with DV index > 0	Median LR Index	IQR	Z-score	P-value

L	11	−0.024	0.177	−0.857	0.391
R	11	−0.022	0.169	−1.057	0.290
L > R	14	0.013	0.095	0.086	0.932
R > L	10	−0.026	0.126	−1.000	0.317
Dorsal Third Digit					
Contrast	Number of subjects with DV index > 0	Median LR Index	IQR	Z-score	P-value

L	14	0.024	0.145	0.171	0.864
R	11	−0.015	0.130	−0.486	0.627
L > R	7	−0.059	0.195	−1.971	**0.049**
R > L	5	−0.092	0.158	−3.229	**0.001**

Bold = p < 0.05. L = left, R = right, D = dorsal, V = ventral, IQR = interquartile range.

## Abbreviations

MRI: Magnetic resonance imaging
fMRI: Functional magnetic resonance imaging
BOLD: Blood oxygenation level dependent
LR: Left-right
DV: Dorsal-ventral
COG: Center-of-gravity
TSNR: Temporal signal-to-noise ratio
