# Supplementary material for: Assessing the spatial distribution of cervical spinal cord activity during tactile stimulation of the upper extremity in humans with functional magnetic resonance imaging
Source: Neuroimage. Author manuscript; Available in PMC 2020 Aug 15. (PMC7386934; doi:10.1016/j.neuroimage.2020.116905)
Supplement: 1 [file NIHMS1608981-supplement-1.pdf]

Assessing the Spatial Distribution of Cervical Spinal Cord Activity during Tactile Stimulation of the Upper Extremity in Humans with Functional Magnetic Resonance Imaging

Kenneth A. Weber II, Yufen Chen, Monica Paliwal, Christine S. Law, Benjamin S. Hopkins, Sean Mackey, Yasin Dhaher, Todd B. Parrish, and Zachary A. Smith

Supplementary Material

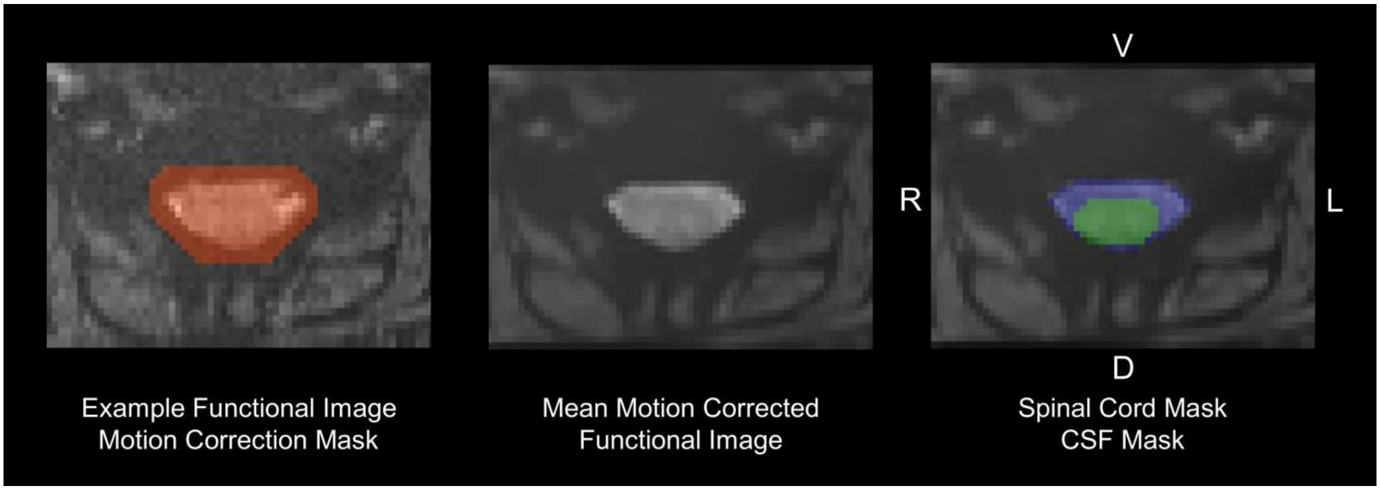

Supplementary Fig. 1. Example motion correction, spinal cord, and cerebrospinal fluid (CSF) masks. Motion correction was performed using a manually drawn binary mask (orange) to exclude areas of non-rigid motion outside the spinal column. A spinal cord mask (green) and CSF mask (blue) were also manually drawn using the mean motion corrected functional image. The spinal cord mask was used for spatial normalization of the functional images to standard space. The CSF mask was used to generate slice specific CSF regressors, which were used for denoising the functional time series. Axial slices from a representative participant are shown. D = dorsal, V = ventral, L = left, R = right.

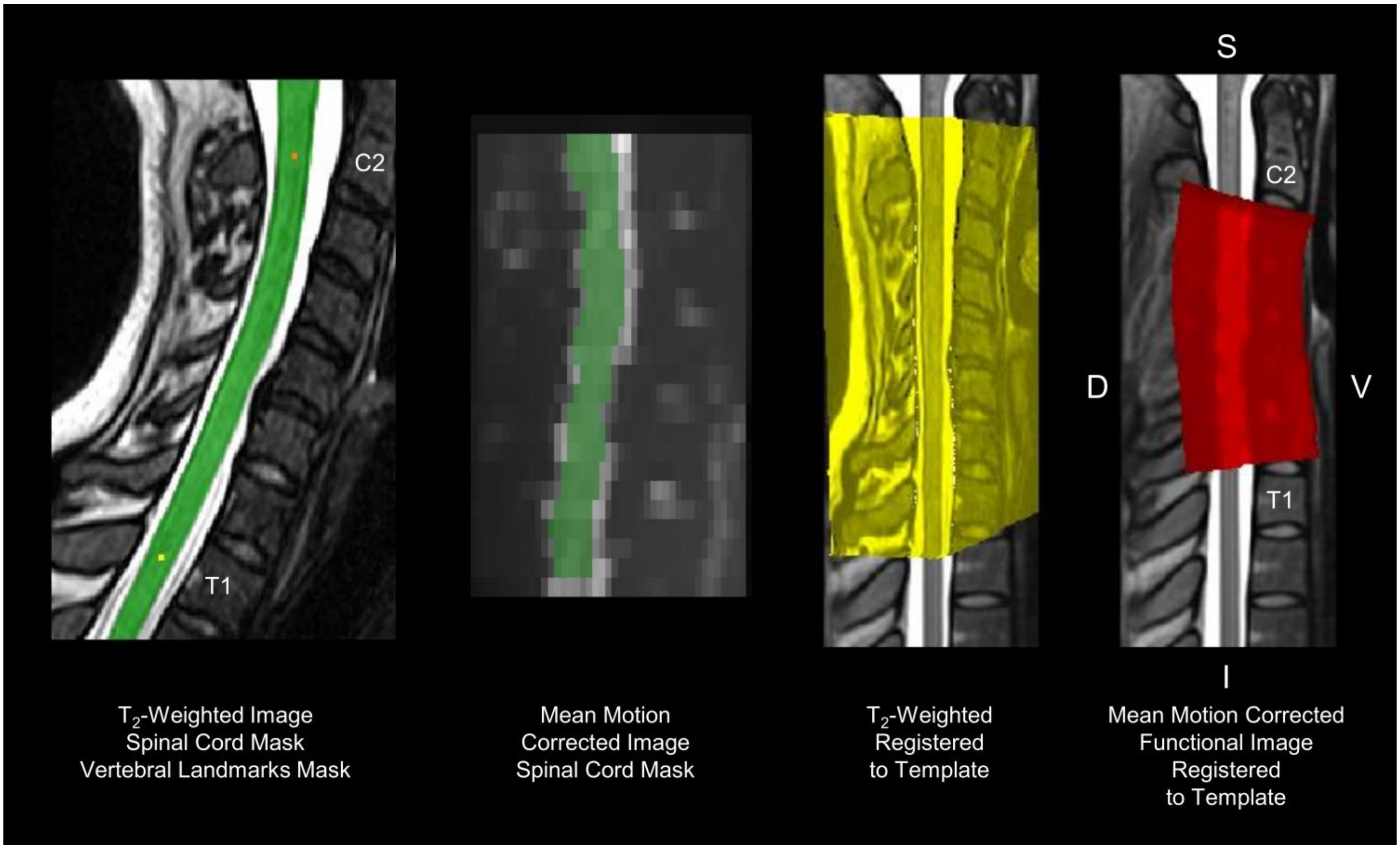

Supplementary Fig. 2. Spatial normalization of the functional time series to the PAM50 template. Spatial normalization is the process of bringing the subject level images into a common space. The T_2-weighted structural image (effective resolution = $0.8 \times 0.8 \times 0.8$ mm^3, interpolated resolution = $0.8 \times 0.4 \times 0.4$ mm^3) was first non-linearly registered to the PAM50 template (resolution = $0.5 \times 0.5 \times 0.5$ mm^3) using the spinal cord (green) and manually generated vertebral landmarks (C2 and T1 vertebral levels) masks. The spinal cord was then manually segmented from the mean motion corrected functional image, and the PAM50 T_2*-weighted template was then registered to the mean motion corrected functional image using the template to structural warping field and the spinal cord mask (green) to initialize the registration. The warping fields from each step of the normalization process were then concatenated allowing for the transformation of the functional images to standard space. Sagittal slices from a representative participant are shown. The T_2-weighted structural image (yellow) and the mean motion corrected functional image (red) warped to template space are shown overlaid the PAM50 T_2-weighted spinal cord template. S = superior, I = inferior, D = dorsal, V = ventral.

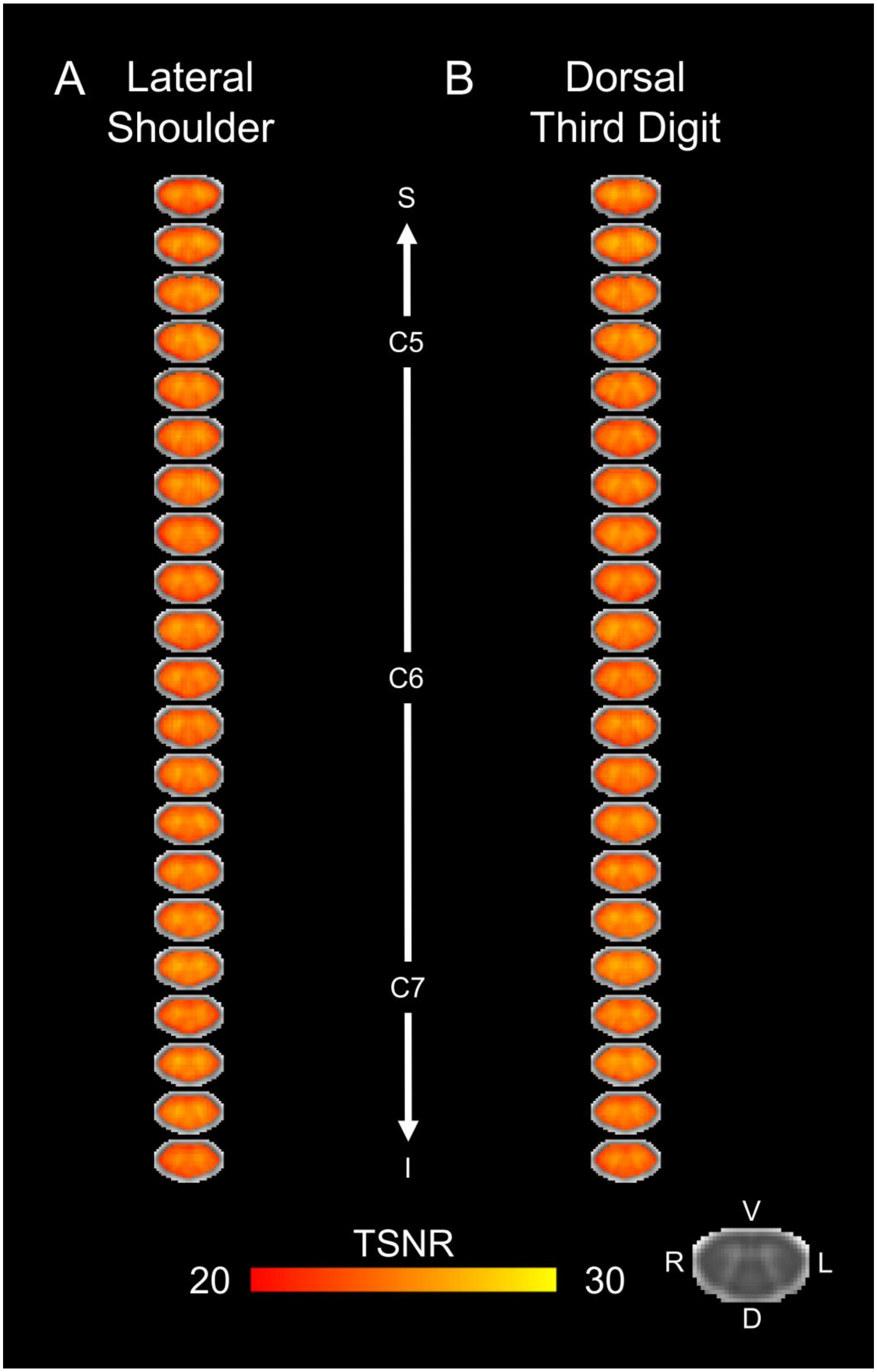

Supplementary Fig. 3. Temporal signal-to-noise (TSNR) maps for the lateral shoulder (A) and dorsal third digit stimulation (B) runs following motion correction, slice-timing correction, temporal filtering, and normalization to standard space. TSNR is a measure of the stability of the signal over the functional time series. The approximate locations of the centers of the C5, C6, and C7 spinal cord segments are shown. Every 4th axial slice from the intersection of the subject level functional images is shown. The background image is the PAM50 T_2^*-weighted spinal cord template. S = superior, I = inferior, D = dorsal, V = ventral, L = left, R = right.

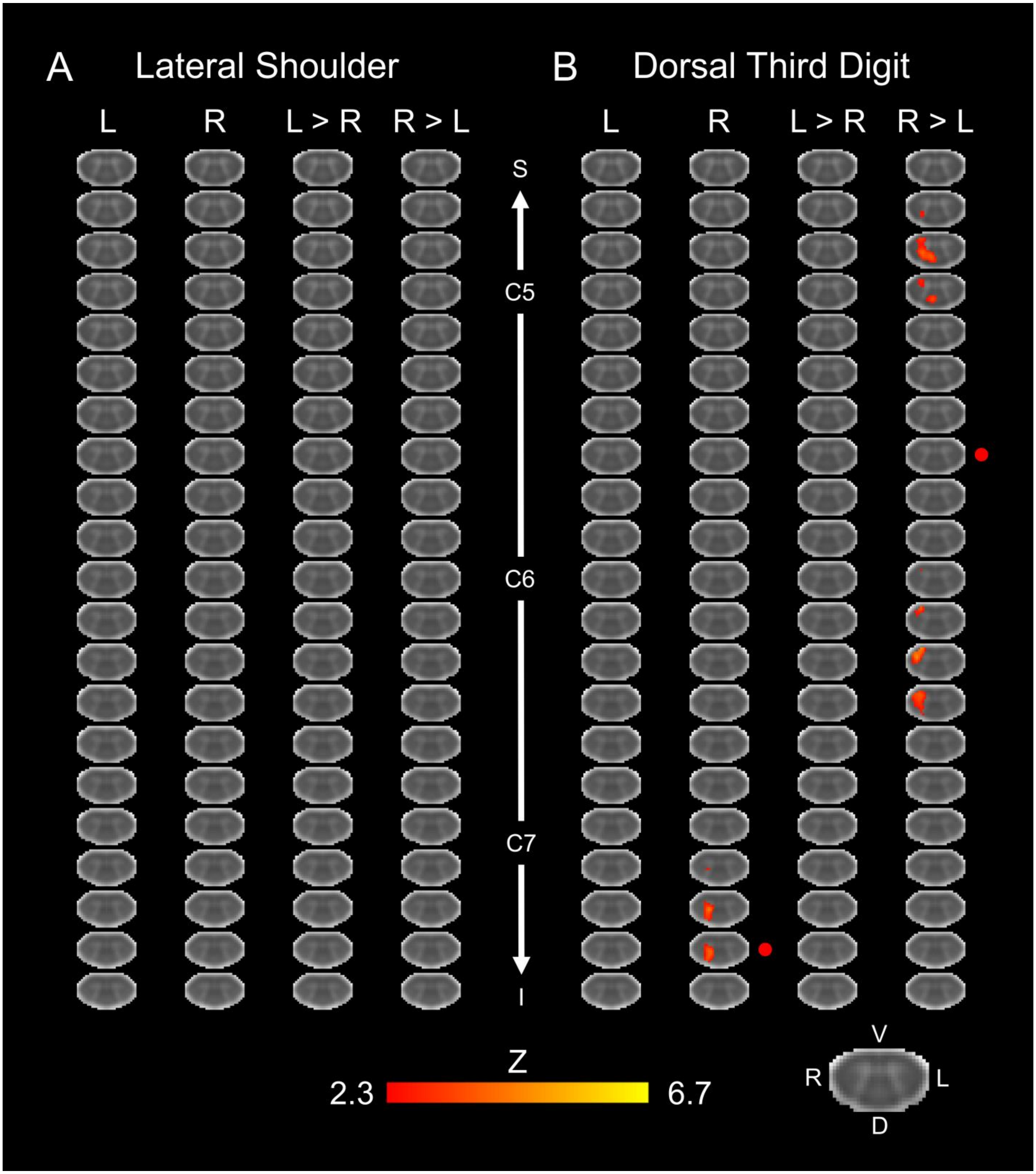

Supplementary Fig. 4. Mixed effects group level activity for the lateral shoulder (A) and dorsal third digit stimulation (B) for the left (L), right (R), and L > R, and R > L stimulation contrasts. Only significant activity was present for the dorsal third digit stimulation for the R and R > L contrasts. The approximate locations of the centers of the C5, C6, and C7 spinal cord segments are shown. Every 4th axial slice from the intersection of the subject level functional images is shown. The red circles indicate the approximate center-of-gravity of the activity along the superior-inferior axis. The activation maps were generated using a voxel-wise thresholded at a Z-score > 2.3 with a cluster-level corrected threshold of $p < 0.05$. The background image is the PAM50 T_2^*-weighted spinal cord template. S = superior, I = inferior, D = dorsal, V = ventral, L = left, R = right.

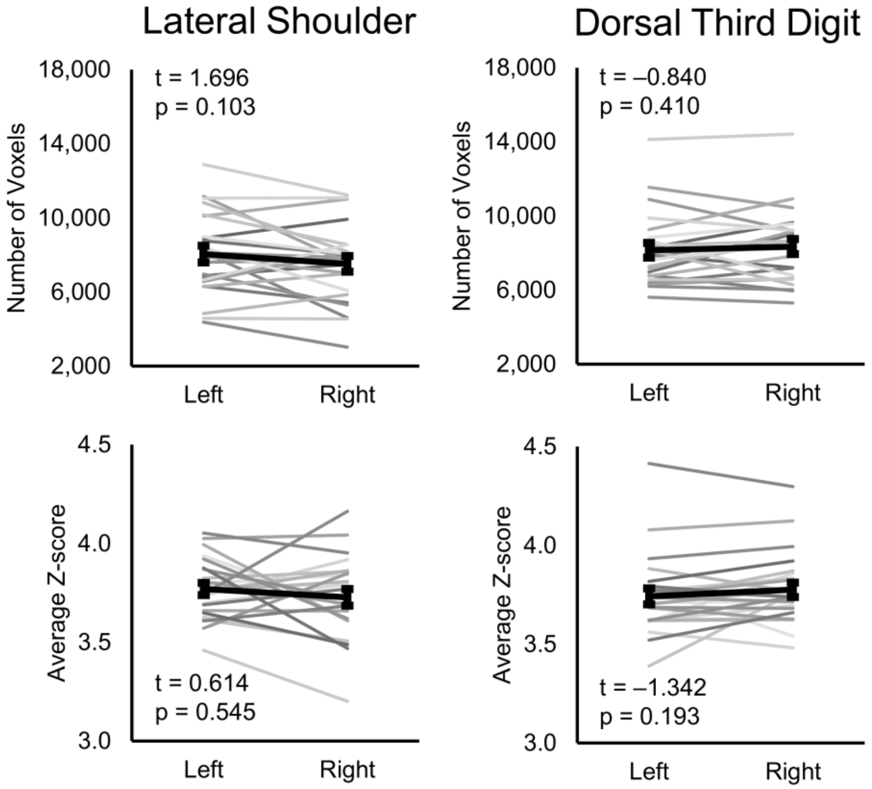

Supplementary Fig. 5. Comparing the number of active voxels and average Z-score of the active voxels at the subject level for the lateral shoulder and dorsal third digit stimulation between the left- and right-sided stimuli. Subject level activity was defined using a voxel-wise threshold of Z-score > 2.3 with no correction for multiple comparisons. The average number of active voxels and the average Z-score of the active voxels across the participants are shown in black. Error bars = ± standard error.

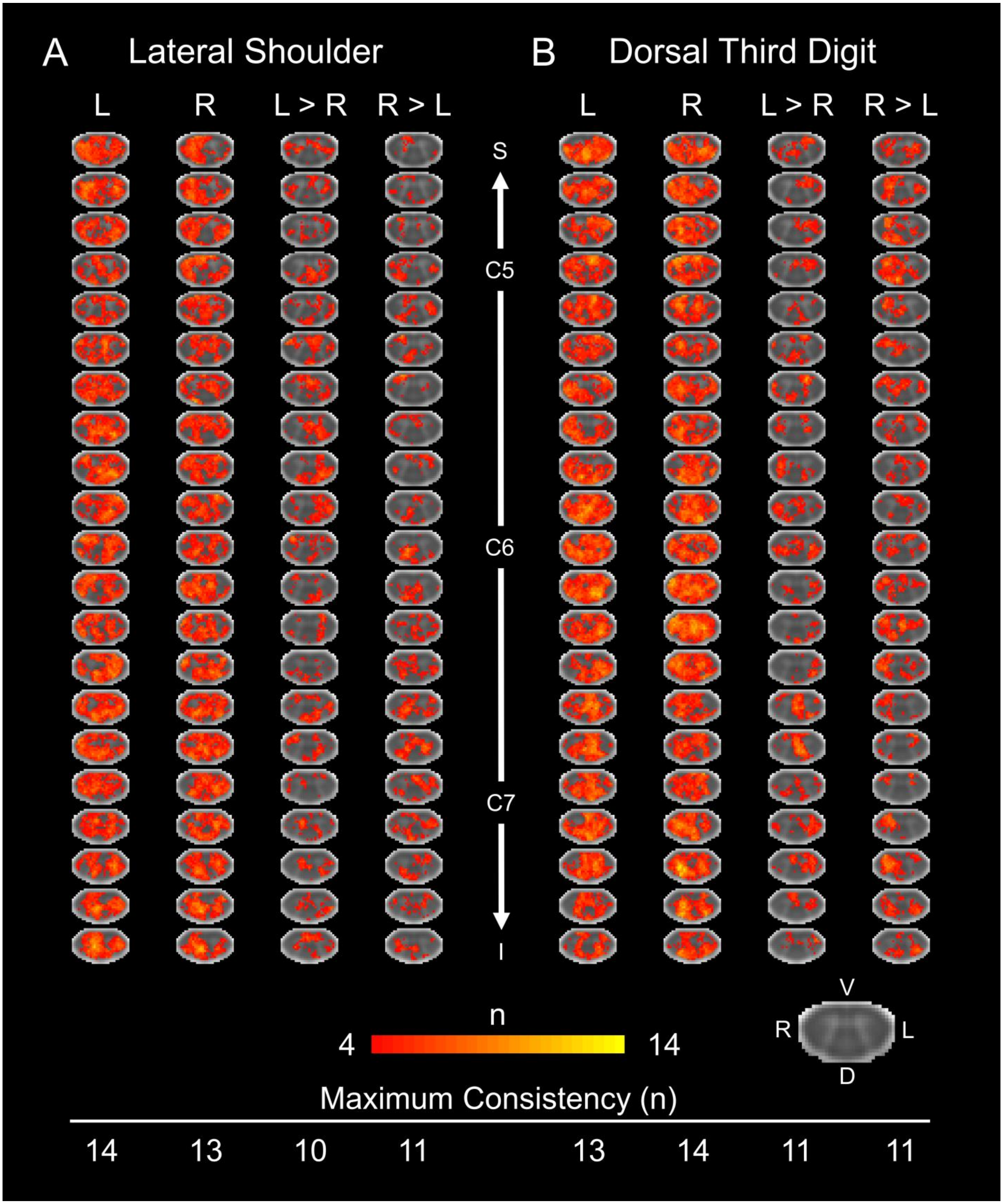

Supplementary Fig. 6. Subject level consistency maps for the lateral shoulder (A) and dorsal third digit stimulation (B) for the left (L), right (R), and L > R, and R > L stimulation contrasts. The consistency maps show the number of participants with activity. The maximum overlap across the maps was only 14 of the 24 participants, suggesting considerable intersubject variability in the spatial localization of the activity across the spinal cord. The approximate locations of the centers of the C5, C6, and C7 spinal cord segments are shown. Every 4th axial slice from the intersection of the subject level functional images is shown. Subject level activity

was defined using a voxel-wise threshold of Z-score > 2.3 with no correction for multiple comparisons. The background image is the PAM50 T_2^*-weighted spinal cord template. S = superior, I = inferior, D = dorsal, V = ventral, L = left, R = right.

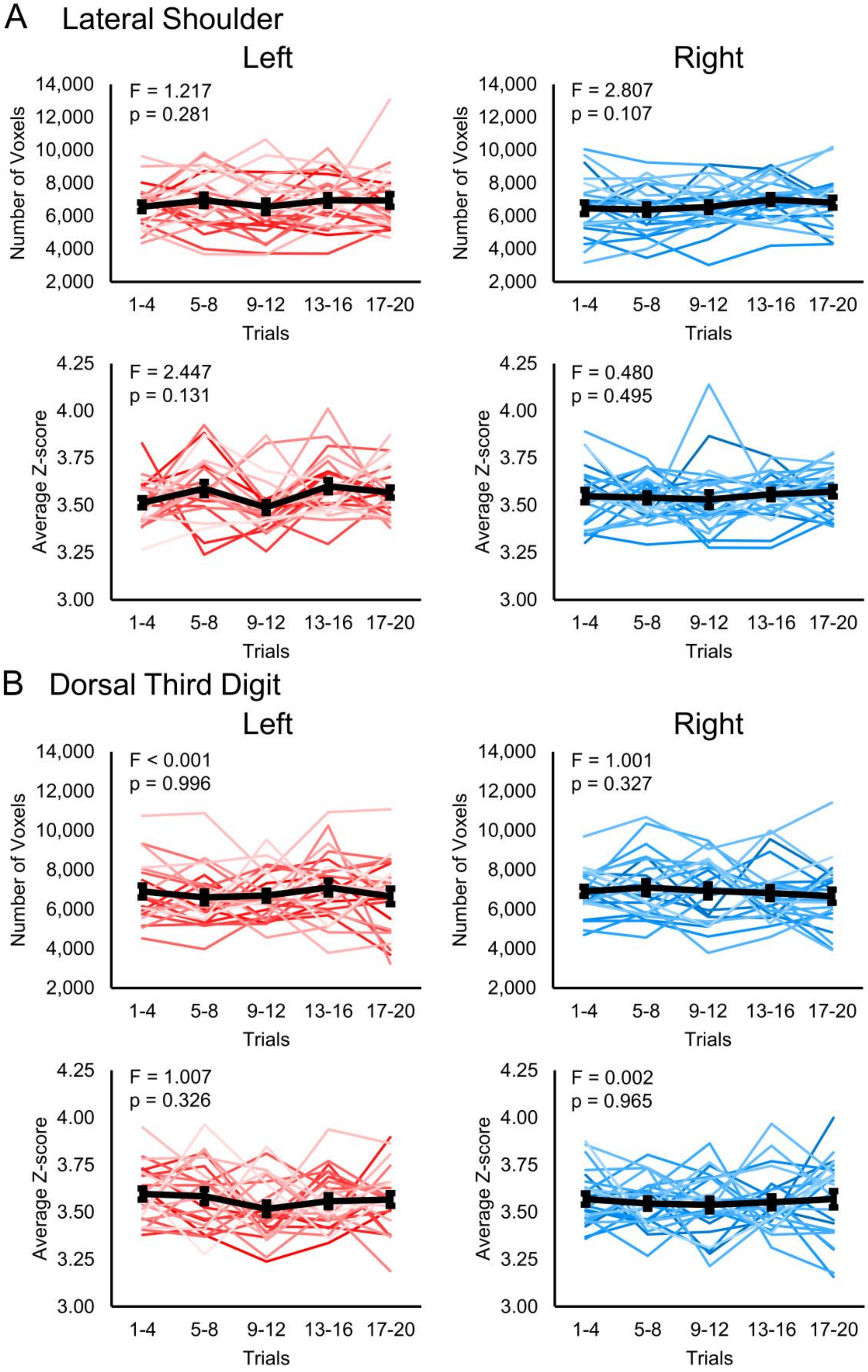

Supplementary Fig. 7. Subject level time-dependent changes in the number of active voxels and average Z-score of the active voxels for the lateral shoulder (A) and dorsal third digit (B) stimulation. To investigate time-dependent changes, the stimulation run was divided into five sets of four consecutive trials. The average number of active voxels and the average Z-score of the active voxels for each set were averaged for each stimulation

site to assess any time-dependent changes possibly due to habituation or sensitization to the repeated stimuli. No significant changes over time were identified. The average number of active voxels and the average Z-score of the active voxels across the participants are shown in black. No significant time-dependent linear increases or decreases in the number of active voxels or the average Z-score of the active voxels were present (repeated measures ANOVA with linear contrast). Error bars = ± standard error.
